# Multifunctional Single-Phase Photocatalysts: Extended Near Infrared Photoactivity and Reliable Magnetic Recyclability

**DOI:** 10.1038/srep15511

**Published:** 2015-10-27

**Authors:** Xiaoning Li, Zhu Zhu, Feng Li, Yan Huang, Xiang Hu, Haoliang Huang, Ranran Peng, XiaoFang Zhai, Zhengping Fu, Yalin Lu

**Affiliations:** 1 CAS Key Laboratory of Materials for Energy Conversion, Department of Materials Science and Engineering, University of Science and Technology of China, Hefei 230026, P. R. China; 2 Hefei National Laboratory for Physical Sciences at Microscale, University of Science and Technology of China, Hefei 230026, P. R. China; 3 Synergetic Innovation Center of Quantum Information & Quantum Physics, University of Science and Technology of China, Hefei 230026, P. R. China; 4 National Synchrotron Radiation Laboratory, University of Science and Technology of China, Hefei 230026, P. R. China; 5 Laser Optics Research Center, US Air Force Academy, Colorado 80840, USA

## Abstract

A practical photocatalyst should be able to integrate together various functions including the extended solar conversion, a feasible and economic recyclability, and above the room temperature operation potential, *et al.*, in order to fulfill the spreading application needs in nowadays. In this report, a multifunctional single-phase photocatalyst which possesses a high photoactivity extended into the near infrared region, an easy magnetic recyclability and the high temperature stability was developed by doping Co into a new layer-structured Bi_7_Fe_3_Ti_3_O_21_ material. Light absorption and photocatalytic activity of the resulted Bi_7_Fe_3-x_Co_x_Ti_3_O_21_ photocatalyst were extended to the long wavelength as far as 800 *n*m. Its strong ferromagnetism above the room temperature enables the nanopowders fully recyclable in viscous solutions simply with a magnet bar in an experimental demonstration. Furthermore, such photoactivity and magnetic recyclability were heavily tested under high-temperature and high-viscosity conditions, which was intended to simulate the actual industrial environments. This work brings the bright light to a full availability of a new multifunctional photocatalyst, via integrating the much enhanced ferromagnetic, ferroelectric, optoelectronic properties, most importantly, into a single-phase structure.

Scope of the photocatalytic application has been continuously expanding as the number of polluted aqueous sources abruptly increases in nowadays. Majority of the aqueous wastes discharged directly from common industrial facilities are viscous and usually hot at a temperature above the room temperature. Those photocatalytic applications including water photolysis or water purification, therefore, require the to-be-used photocatalysts to meet a few quite straightforward yet very challenging criteria. Firstly, photocatalysts to be used should have a fairly large extension into the near infrared (NIR) in light absorption, in order to cover as much as possible the solar spectrum and to efficiently convert the solar energy into the chemical energy. Those currently under-the-investigated photoactive materials and their inside micro- or nano-scale structures are also required to be optimized, in order to ensure a lower enough recombination rate of the photon-generated electrons and holes. Secondly, the nanoscale photocatalysts to be used in traditionally pollutant degradation or water photolysis systems should be feasibly and economically recyclable^1^, avoiding the further secondary contamination and reducing the actual implementation cost. In fact, recycling of such nanoscale photocatalysts in waste solutions is an important but extremely difficult task due to their small sizes, high viscosity, and high environmental temperature. That to use a permanent magnetic field should be considered as a natural and economic choice, and this, however, requires the photocatalysts to be strongly magnetically responsive, especially above the room temperature. At last, the above-the-room temperature environment when using such nanoscale photocatalysts requires them remaining photocatalysis highly active^2^, and stable in much harsher conditions too. That to find a new single-phase material able to possess all the above properties to realize the needed multifunctions at the same time, however, is indeed a very challenging task.

For the first criteria discussed above, a few strategies have been investigated in the past. For examples: i) using those materials with narrower band gaps, such as chalcogenides or AgPO_3_^3^,^4^,^5^. However, besides that their stability in water under the solar irradiation is a serious concern, such chalcogenides are usually toxic^6^, ii) anchoring the wide band gap TiO_2_ with other visible-active components, such as metals or dyes^7^. Noble metals of Ag, Au or Pt have been usually used for the metal-anchored plasmon-enhanced photocatalysts. However, they are expensive which limits their large scale usage potential^8^, while the organic dyes normally will decay under the solar irradiation^9^, iii) improving the visible light activities of the wide band gap oxide materials by using dopants^10^,^11^,^12^, or by the disorder engineering^13^,^14^. In fact, the mechanisms via doping to induce the visible extension in most cases still need to be uncovered^15^, and the doping itself may generate more unwanted carrier recombination centers. Furthermore, thermal stability of such doped materials requests more studies to fulfill their long term service goal^16^,^17^, iv) One-dimensional (1D) nanostructures have gained attention in solar energy conversion because they have a long axis to absorb incident sunlight yet a short radial distance for separation of photogenerated charge carriers^18^,^19^. For the second criteria discussed above, nanoscale heterostructuring the photoactive materials has been receiving attracting interests in recent years, due to the convenience of integrating together both magnetism and photoactivity simply by using two separate materials^20^,^21^,^22^,^23^. However, properties of such heterostructured nanomaterials, normally with a magnetic core of Fe_3_O_4_ (easily oxidized in air) or γ-Fe_2_O_3_ (easily undergoing a phase transformation to paramagnetic α-Fe_2_O_3_), are less stable^22^,^24^. Therefore, new photocatalysts with a single-phase structure, possessing an excellent combination of good NIR activity, magnetic recyclability, high photocatalysis efficiency, and the potential able to be used in harsh conditions and at or above the room temperature, are highly in demand. Unfortunately, such single-phase and multifunctional photocatalysts (MP) have not been well explored in the past, though that this indeed has been becoming a new trend in the effort of searching for the next generation photocatalysts.

That to use the materials (Bi_2_O_2_)(A_m−1_B_m_O_3m+1_) with layered structures as potential multifunctional photocatalysts can be promising. With hopes and in theory, photocatalysis activities in such materials could be feasibly modulated by varying dopants, or by changing their layer structures. Unfortunately, pursuing multifunctions in such materials as new multifunctional photocatalysts has been hindered, mainly due to the strong challenges in realizing their room temperature ferromagnetism (FM), NIR spectrum coverage, and importantly, the fabrication of their single-phase yet nanoscale materials which should be stayed stable enough for actual field implementation. In fact, each one of such challenges is serious enough to researchers in the areas of multiferroics and optoelectronics, and breakthroughs in such areas are highly expected.

When considering using cobalt and iron incorporated 6-layer Bi_7_Fe_3−x_Co_x_Ti_3_O_21_ (BFCTOs) as a potential MP, the anticipated integrated multiple functions could be realized based on the following analyses. Firstly, inserting both Fe and Co elements together could decrease the resulting material’s band gap. According to the past work of the first principle calculations of 3-layer Aurivillius Bi_4_Ti_3_O_12_ and 4-layer Bi_5_FeTi_3_O_15_, their band structures are similar to that of TiO_2_, i.e., the valence band is composed of states with predominant O-2*p* character and the conduction band with mainly the Ti-3*d* character, while the contribution of Bi states to the band edge could be neglected^25^,^26^. When the transition metal elements being incorporated into TiO_2_ or SrTiO_3_, the Co-3*d* states will overlap with the Ti-3*d* states and this lowers the conduction band edge, while the Fe-states will overlap with O-2*p* states and then raises the valence band edge, which further decreases the band gap^27^,^28^. Limited by the current computation capability, an accurate calculation of the electronic structures of BFCTO via the first principle calculation would be difficult. It should be reasonable, however, to assume its analogousness to TiO_2_, and in fact, this assumption was partially confirmed by the past experiments in Bi_4_Ti_3_O_12_^29^,^30^. Secondly, the exchange interaction between Fe and Co ions via bonding with oxygen, as well as the spin canting via the antisymmetric Dzyaloshinskii–Moriya (DM) interaction, could give rise to a much enhanced room temperature ferromagnetic in such layered oxides^31^. Furthermore, radius differences between the introduced Co and Fe ions inside the crystalline structures could lead to a more favorable realization of the polar ground states and then generate a strong ferroelectricity (FE)^32^. In fact, a remarkable coexistence of both FE and FM well above RT by substituting both Fe with Co into a 4-layered material has been demonstrated^33^,^34^,^35^,^36^,^37^, which indicates a breakthrough in the area of multiferroic materials. This much enhanced FM could be used for the anticipated RT magnetic recyclability in MPs.

With above analyses and in this work, nanoplates of the layer-structured Bi_7_Fe_3-*x*_Co_*x*_Ti_3_O_21_ (with *x* = 0.1, 0.2, 0.25) were fabricated by a facile hydrothermal method. The resulting nanoplates were experimentally demonstrated as a new single-phase photocatalyst with anticipated multifunctions, of which the light absorption was successfully extended to the NIR as far as 800 *n*m, and the enhanced room temperature ferromagnetic character made them feasibly and efficiently recyclable in viscous solutions simply by a magnet bar in the laboratory demonstrations. Furthermore, stability of the photocatalysis and the magnetically recyclability of such nanoplates were also reaffirmed in the well above the room temperature condition (~343K), which brings a greater hope for using such MPs for practical applications.

## Results and Discussions

### Structure and morphology

**Figure 1:**
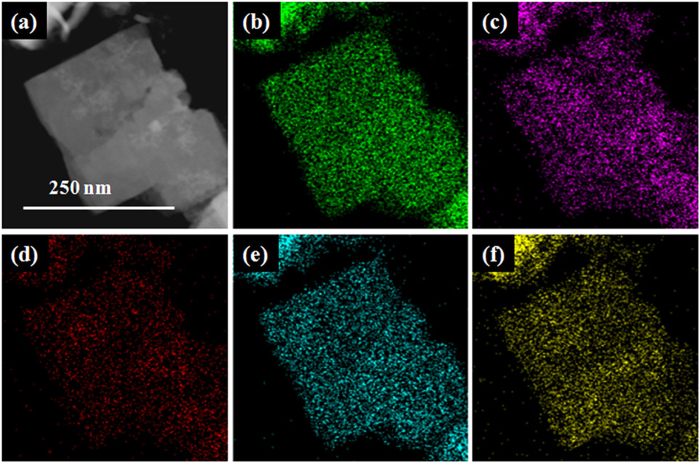
STEM image and the corresponding EDS elemental mapping of the nanoplate-like BFCTO-0.25 (a) STEM, (b) Bi, (c) Ti, (d) Fe, (e) Co and (f) O elements.

All BFCTO samples were prepared by the single-step hydrothermal method (Fig. S1). The products were nanoplate-like with the thickness of 30–60 *n*m, as demonstrated by the SEM and TEM images in Fig. S2. Crystallinity of the nanoplates was inspected by the selected area electron diffraction (SAED) pattern in inset of Fig. S2h, which could be well indexed to Bi_7_Fe_3_Ti_3_O_21_ (a space group of F2*mm* (42), JCPDS 54–1044), consistent with the powder XRD pattern in Fig. S3. To study the elemental distribution of the BFCTOs nanoplates, STEM images and the corresponding EDS elemental mapping of the BFCTO-0.25 sample are shown in Fig. 1. Homogeneous distribution of Bi, Ti, Fe, Co and O elements was observed inside the nanoplate, implying a successful synthesis of the BFCTO with a uniform layered structure. XPS measurements were carried out to obtain additional electronic structural information regarding the samples. The fitted spectra were shown in Fig. S4 and binding energy together with valance state were shown in Table S1, from which we can draw the conclusion that Bi ion is with +3, Fe ion +3, Ti +4, and Co +3 (only for BFCTOs) respectively, consistent with the formula Bi_7_Fe_3-*x*_Co_*x*_Ti_3_O_21_. Atomic configuration from the HRTEM of a BFCTO-0.25 nanoplate in Fig. S5 coincided with the calculated crystalline structure of Bi_7_Fe_3_Ti_3_O_21_ (also with a space group of F2*mm* (42), JCPDS 54–1044), which further implied that the crystalline structure of BFCTOs is the same with that of BFTO when the doped cobalt content is low. Co and Fe preferred to occupy Ti sites due to the similarity of the ionic radius between them^33^,^34^,^35^,^36^,^37^. Though an increase of Co may further improves the materials FM properties, high cobalt concentration may lead to the variation of the resulted lattice structures, which in fact was manifested that the peak position shifts when x > 0.25, as shown in Fig. S3.

### Ferroelectric, ferromagnetic and optical properties

**Figure 2:**
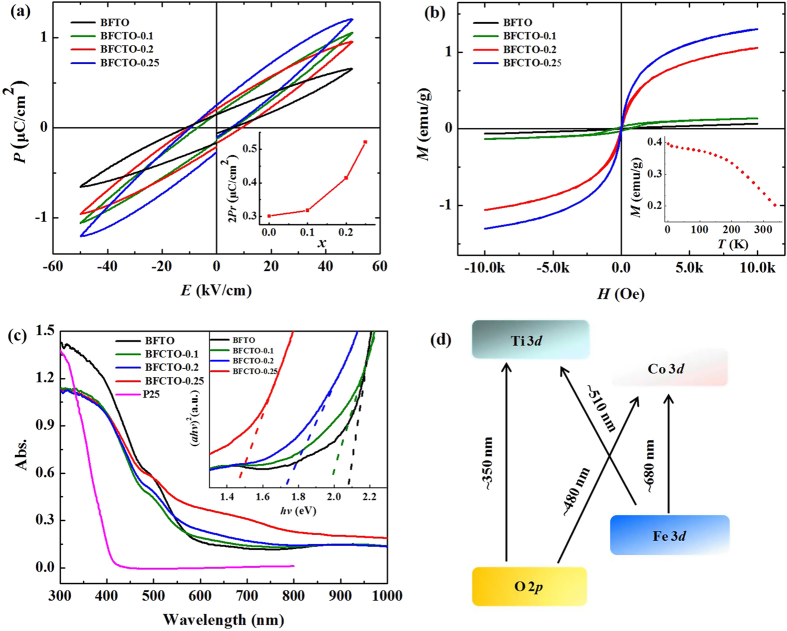
Multiferroic and optical properties of BFCTOs. (**a**) P-E hysteresis loops of different photocatalysts; inset is variation of 2P_r_ with Co content x; (**b**) M-H hysteresis loops of different photocatalysts at RT; inset is a temperature dependence of the magnetization for BFCTO-0.25 with an applied magnetic field set at 200 *Oe*; (**c**) UV-Vis diffuse reflectance spectra, inset is the plot of (*ahv*)^2^ versus photon energy *hv* used to calculate the *E*g value; (**d**) Illustration of enhanced light absorption due to transition metal 3*d* state.

The spontaneous internal electric field originated from the ferroelectricity has been reported to help a better separating of electrons and holes^38^,^39^,^40^. Here P-E loops of the un-sintered pellets composed of pressed BFCTOs at RT were measured. As shown in Fig. 2a, the P-E measurement exhibited well defined hysteresis loops without reaching the saturation. The saturation should be achieved if the applied electric field is large enough, however, this may be limited by the current leakage. Inset of Fig. 2a is a variation of 2P_r_ with Co content x, showing that the residual polarization of BFCTOs was enhanced with an increase of the Co content. Magnetic hysteresis loops (M-H) of BFCTOs powders at RT are displayed in Fig. 2b. All BFCTO samples presented well defined hysteresis loops while the BFTO (without Co) is almost paramagnetic, that is to say, BFCTOs become ferromagnetic when doped with Co, which was further confirmed by the zero-field cooling (ZFC) and field cooling (FC) curves (Fig. S6). BFCTO-0.25 performed the best ferromagnetism with a saturated magnetization of 2M_s_ = 2.62 *emu*/g, remnant magnetization of 2M_r_ = 0.04 *emu*/g and coercive field 2H_c_ = 36 *Oe* at RT. M-T curve of BFCTO-0.25 was measured in order to characterize the temperature dependent magnetism. As illustrated in the inset of Fig. 2b and Fig. S7, though the ferromagnetism of the sample decreased when the temperature increased, the ferromagnetism could be kept up to 350 K, indicating that the Curie’s temperature is higher than 350 K.

The UV-Vis diffusive reflectance spectra was measured and displayed in Fig. 2c. BFCTOs have a benign absorption beyond 580 *n*m and extended to the NIR wavelength as long as 800 *n*m, contrasted to the commercial Degussa P25^38^. The optical absorption near the band edge follows the Kubelka-Munk function^41^: α*hν* = *A*(*hν* − *E*_g_)^*n*/2^, where α, *h, v, E*_g_, and *A* are the absorption coefficient, Planck constant, light frequency, band gap, and a constant, respectively. The power index *n* depends on the kind of electronic transition, *n* equals to 1 for a direct band-gap material and 4 for an indirect band-gap material. For BFCTOs, *n* is determined to be 1. The plot of (*αhv*)^2^ versus photon energy *hv*, by extrapolating the straight portion of (*αhv*)^2^ against *hv* plot to the point α = 0, as shown in the inset of Fig. 2c. We got the *E*_g_ value as 2.08 eV, 1.97 eV, 1.71 eV and 1.45 eV respectively for Co content increasing from 0 to 0.25. It indicates that doping Co in the BFTO can efficiently reduced the band gap of materials. Similar to Bi_5_FeTi_3_O_15_, the valence band of Bi_7_Fe_3_Ti_3_O_21_ is the mixing of Fe-3*d* band with O-2*p* band, while the conduction band is from Ti-3*d*. The incorporation of Co will lower the conduction band edge by mixing Co-3*d* and Ti-3*d*^27^,^28^. Therefore, the possible inter band transitions and the corresponding light absorption bands are illustrated in Fig. 2d. The absorption band around 480 *n*m might arise from the transition from O-2*p* to Co-3*d*, and that at 510 *n*m is from Fe-3*d* to Ti-3*d*, while the band at 680 *n*m is from Fe-3*d* to Co-3*d*^42^,^43^,^44^,^45^.

### Photocatalysis under visible light

**Figure 3:**
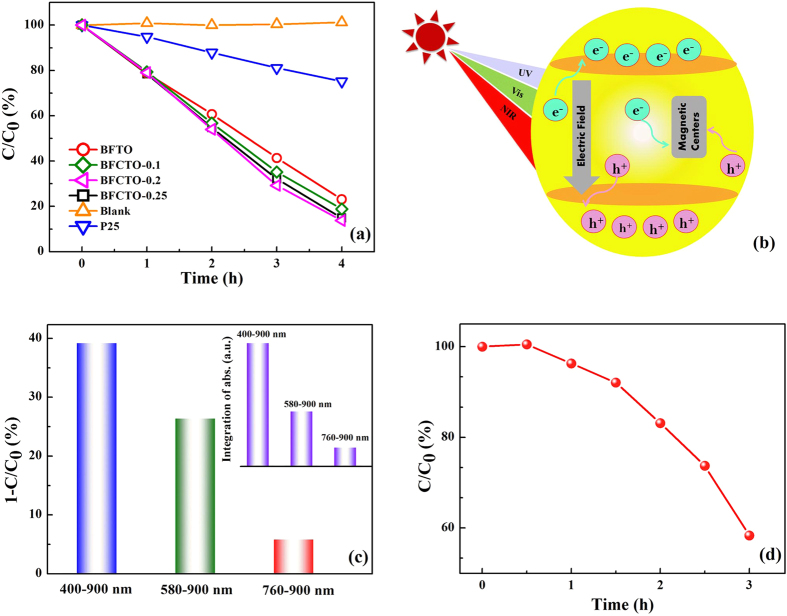
Photocatalysis under visible light and NIR light irradiation. (**a**) Amounts of the decomposed RhB under a 20 W fluorescent lamp light (400–760 *n*m) when using different photocatalysts; (**b**) A possible role of ferroelectricity and magnetism for the photocatalysis process; (**c**) Amounts of the decomposed RhB by BFTO and BFCTO-0.25 under a condition that a 400/580/760 *n*m long-wave-pass filter was placed next to a halogen reflector lamp (400 ~ 900 *n*m); inset is the integrated light absorption of BFCTO-0.25 in different wavelength ranges; (**d**) the full visible light photocatalytic degradation of BFCTO-0.25 (50 *m*g)/RhB (5 *m*g/L, 50 *m*L) of 3 hours at 343 K.

In the regard of the actual photocatalysis application, the source of light will be the natural sunlight or an indoor light which are normally weak. In our cases here, photocatalytic activities of the BFCTO powders were tested under a weak visible light source (with only a 20 W fluorescent lamp covering the 400–760 *n*m range), which is significantly lower than those in most reported past experiments. According to the Lambert-Beer theory, the concentration of RhB in the solution should be proportional to the characteristic adsorption peak. Therefore we monitored the absorption peak evolution during the photodegradation of RhB on the base of its maximum absorbance at 554 *n*m from the UV-Vis absorption spectrum (see Fig. S8) and then corresponding plots of C/C_0_ dependence on the irradiation time were drawn in Fig. 3a. The results show that more than 80% of RhB pollutant in the original 5 mg/L solution was decomposed with the 4-hour irradiation. A complete decomposition of RhB was realized with the 6-hour irradiation. To verify that the RhB dye has been degraded instead of just being decolorized, infrared spectra of the primary RhB solution, the RhB solution after the 6-hour photocatalytic reaction, the BFCTOs powders were taken by mixing them into KBr pellets accordingly (Fig. S9). The characteristic absorption bands of RhB among 500 cm^−1^ ~ 1700 cm^−1^ were not observed in the 6-hour solution, as well as that the spectrum of the 6-hour solution is almost identical to that of pure KBr pellet, indicating that there is no organic left in the 6-hour solution. These results strongly support that RhB was fully degraded by the photocatalysis reaction. As the efficiency of the experimental photocatalysis should relate to the light intensity and so on, it would be flimsy to compare our BFCTOs with other reported results using different installations or irradiations, especially different incident powers. In this work, we compared our results with a 25% decomposing efficiency of P25 at the same testing condition (50 *m*g photocatalyst, 50 *m*L 5 *m*g/L RhB, a 20 W visible-light fluorescent lamp, and 4 hours period). Decomposing efficiencies of BFCTOs could be more striking if we further considered the fact that the BET surface areas of all BFCTOs are only ~10 m^2^/g (Table S2), while that of used P25 is ~49.91 m^2^/g.

Though introducing Co into Bi_7_Fe_3_Ti_3_O_21_ can enhance the visible light absorption and the ferroelectricity, the photocatalysis efficiency was not apparently improved with the increasing of Co content. The entire photocatalysis efficiency should be an overall effect of multiple steps such as carrier generation, recombination, migration and the surface degradation reaction, etc. Furthermore, the existence of magnetism may also weaken the photocatalysis activity^22^, as well as that the improved spontaneous polarization will induce aggregation and slightly decrease the specific surface area (Table S2). Therefore, as a result of the competition among all involved processes, photocatalysis efficiencies of the tested samples are in fact all similar while the one from BFCTO-0.2 is only slightly better.

Finally, the photocatalysis at a high ~343 K (~40 degrees high than the room temperature) was also performed in order to simulate the actual industrial waste situations. The results were displayed in Fig. 3d. Obviously, the photocatalysts still work fine, however, with a lower efficiency than that at the RT, which may be caused by the evaporation induced solvent reduction as well as the light scattering caused by the vaporized water steams which reduces the incident powers.

### Photocatalysis under near infrared light

By doping Co into BFTO, the light absorption cutoff was extended from 560 *n*m to 800 *n*m, and this may help the photocatalysis ability at NIR. In order to verify this, photocatalytic activity in different light regions, especially that under the NIR light was conducted for BFCTO-0.25 by applying a series of long-wave-pass filters. The results are shown in Fig. 3c. After 6 hours of the irradiation, the efficiency was 26% for BFCTO-0.25 when using a 580 nm long-wave-pass filter and 5.8% when irradiated under the 760–900 nm light. Contrastingly, the corresponding value was about 1% for BFTO under the 760–900 nm light, suggesting that doping Co indeed extended the BFTO light responsive spectrum to NIR range. It should be mentioned that irradiation power in this wavelength range is very lower when comparing to those in previous works^41^^,46,47^. (Table S3 gave a careful list of the performances of recently developed NIR-responisve photocatalysts.) Therefore at this low incident power, the NIR photodegradation efficiency in this work is considerably high. Further to verify that the photocatalysis was indeed induced by the absorbed NIR light, the absorption in corresponding NIR wavelength range was integrated according to Fig. 2c. As shown in Fig. 3c and the inset, the photocatalysis efficiency is proportional to the integrated absorption at the corresponding NIR wavelength range, suggesting the fact that though the power of the incident NIR photon is low, they can still be very effective to stimulate the photodegradation process. This may be promoted by the internal electric field.^48^

### Magnetically Retrievable Experiments

**Figure 4:**
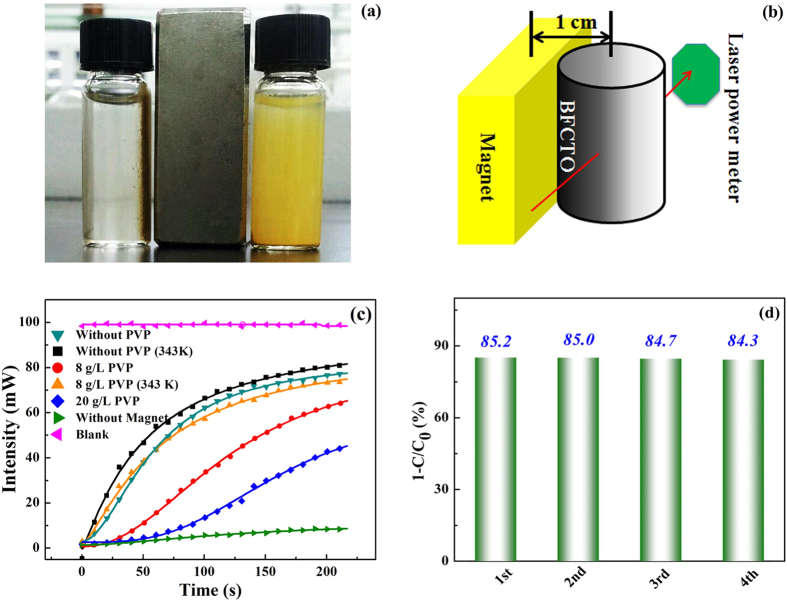
Magnetically Retrievable Experiments of BFCTO-0.25 . (**a**) Photograph of BFCTO-0.25 (right) and BFTO (left) placed nearby a bar magnet after 4 min; (**b**) Sketch of to monitor the light transmittance; (**c**) Dependence of the transmittance intensity of the 980 nm laser light through the BFCTO-0.25 suspension while the magnetic particles moving in the solution under this magnetic bar; (**d**) Cycling experiment of a typical full visible light photocatalytic degradation of BFCTO-0.25 (50 *m*g)/RhB (5 *m*g/L, 50 *m*L) in 4 hours.

Utilizing the strong magnetism at RT, we separated BFCTO-0.25 powders from the suspension by a bar magnet with a low magnetic field strength of ~0.5 T, illustrated in Fig. 4a. The BFCTO-0.25 particles were attracted and agglomerated near the magnet, and the muddy suspension became clear and transparent. As a comparison, the suspension with the BFTO nanoparticles (very weak magnetism at RT) still kept muddy under the same magnetic field. Effect of the magnetic field on the BFCTO-0.25 suspension was further digitized by monitoring the light transmittance of the suspension with a 980 *n*m laser light (the installation see Fig. 4b). As seen in Fig. 4c, the transmittance increases with the time after the magnetic field was applied, and it saturated at about 4 min at RT. When without the magnet, the transmittance increases very slightly with the time if only with the gravity.

In practical applications, the industrial waste solutions are usually hot or sometimes viscous. Here the recycling tests were then conducted using hot and viscous suspensions. Polyvinyl pyrrolidone (PVP K30) with a concentration of 8, 20 *g*/L were added into the BFCTO-0.25 water suspension. The retrieving of the BFCTO-0.25 particles by the magnetic field at RT became slow when increasing the viscosity (Fig. 4c), while the recycling is still acceptable. Recycling at a high temperature of 343 K presented a better performance, probably due to a decrease in the solution viscosity and an increase in particle motion with the magnetism under the high temperature (Fig. 4c). Taking the advantage of this retrievable function, a cycling test was also performed. After the 4 hours reaction, the BFCTO-0.25 photocatalyst was retrieved with the bar magnet, and then the activity of the retrieved photocatalyst was studied again. Results in Fig. 4d suggested that the photocatalytic activity was maintained after several cycles. This shows the BFCTOs with a good stability even at harsh conditions.

## Conclusions

Nanoplate-like Bi_7_Fe_3−x_Co_x_Ti_3_O_21_ (x = 0.1, 0.2, 0.25) were synthesized and tested as stable single-phase multifucntional photocatalysts with the photocatalytic spectral response extended to ~800 *n*m, together with the enhanced ferroelectric and magnetic properties at RT. Photocatalytic activities under both visible light and NIR light were studied and the influence factors were discussed in details. Recycling experiments of the photocatalysts by a bar magnet under different viscosities and at high temperatures were conducted, and the results indicated the material’s great potential as an efficient, single-phase photocatalysts with multifunctions. This work brings the light to improve the solar energy utilization, practical application of the new photocatalysts in the environment management, when carrying forward ferromagnetic, ferroelectric, optical properties into a single-phase structure.

### Experimental section

In a typical synthesis of the Bi_7_Fe_3-x_Co_x_Ti_3_O_21_ nanoplates, Ti(OC_4_H_9_)_4_ (>99.7%), Bi(NO_3_)_3_·5H_2_O (>99%), Fe(NO_3_)_3_·9H_2_O (>98.5%) and Co(NO_3_)_3_·6H_2_O (>99%) were dissolved into HNO_3_ solution, with amounts in the stoichiometric ratio. After a 20 min. of magnetic stirring, the homogeneous metal-ions solution was added into the concentrated NaOH solution. Afterwards, the resulting slurry was transferred into a Teflon-lined stainless steel autoclave (up to ~80% of the total volume). The autoclave was sealed and heated at 200 °C for 2 days, then allowed it to cool down to RT naturally. The sediment was washed with water and ethanol several times and then dried at 60 °C for 8 h.

Purity and crystallinity of the as-prepared samples were characterized by X-Ray powder diffraction (XRD) with the patterns recorded on a Rigaku-TTR III X-ray diffractometer with the Cu-Kα radiation. X-ray photoelectron spectroscopy (XPS) analyses were performed using an ESCALAB 250 system (Thermo Scientific). Morphologies of the powders were observed by a scanning electron microscopy (SEM, JSM-6700F) and a transmission electron microscopy (HRTEM, JEM-2010). Scanning transmission electron microscope (STEM) and elemental mapping were conducted with a field-emission transmission electron microscope (FETEM, JEOL JEM-2100F) equipped with an energy dispersive X-ray spectroscopy (EDS). UV-Vis-NIR diffusive reflectance spectroscopy of the powders was measured by using a UV-Vis-NIR spectrophotometer (SOLID3700). The Brunauer-Emmett-Teller (BET) surface area was estimated by using the adsorption data (Tristar II 3020M, Mircomeritics, USA). Magnetic properties were characterized by the vibrating sample magnetometer (VSM) option of the Quantum Design physical property measurement system (PPMS) (Quantum Design, USA). The polarization-field (P-E) loops were investigated by a Precision LC ferroelectric analyzer (Radiant Technology Product, USA) on a pressed disk directly from nanomaterials without further sintering (the used pressure is 6 MPa; the disk has a diameter of 12 *m*m and a thickness of 0.5 *m*m; it was coated with silver paste as electrodes with an area of 3.14 *m*m^2^).

Photocatalytic activities under the Vis-NIR irradiation were investigated by photodegradation of RhB under two schemes: (1) a 20 W fluorescent lamp with a full visible light range (400–760 *n*m) was placed 10 cm away from the sample to irradiate 50 *m*L BFCTOs (50 *m*g)/RhB (5 *m*g/L) suspension under a continuous magnetic stirring, the solution was eclipsed in the dark for 1 hour to reach the adsorption-desorption equilibrium between the photocatalyst and RhB before illumination. 4 *m*L suspension was sampled at every 1h interval. After the photocatalysis test, the photocatalyst in the sampled suspension as well as in the remnant suspension were gathered by a magnet bar, and then the photocatalysis test was conducted again with the gathered photocatalyst. (2) The photocatalysis activity in different wavelength regions was investigated by placing a long-wave pass filter (400, 580 or 760 *n*m) between a halogen reflector lamp (420 *n*m–900 *n*m, 50 W) and the BFCTO-0.25/RhB (2 *m*g/L, 25 *m*L) suspension under mechanical agitation, the solution was eclipsed in the dark for 1 hour to reach the adsorption-desorption equilibrium between the photocatalyst and RhB before illumination, the remaining concentration of RhB after the 6 hours irradiation was measured. The concentration of RhB in the solution was estimated on the base of its maximum absorbance at 554 *n*m from the UV-Vis absorption spectrum.

## Additional Information

**How to cite this article**: Li, X. *et al.* Multifunctional Single-Phase Photocatalysts: Extended Near Infrared Photoactivity and Reliable Magnetic Recyclability. *Sci. Rep.* **5**, 15511; doi: 10.1038/srep15511 (2015).

## Supplementary Material

Supplementary Information
