# Supplementary material for: Multifunctional Single-Phase Photocatalysts: Extended Near Infrared Photoactivity and Reliable Magnetic Recyclability
Source: Sci Rep. 2015 Oct 27;5:15511. doi: 10.1038/srep15511 (PMC4621415; doi:10.1038/srep15511)
Supplement: Supplementary Information [file srep15511-s1.doc]

**Multifunctional Single-Phase Photocatalysts:** **Extended Near Infrared Photoactivity and Reliable Magnetic Recyclability**

Xiaoning Li^1^, Zhu Zhu^1^, Feng Li^1^, Yan Huang^1^, Xiang Hu^2^, Haoliang Huang^1^, Ranran Peng^1,3^, XiaoFang Zhai^2,3^, Zhengping Fu^1,3,*^, Yalin Lu^1,2,3,4,5,*^

^1^CAS Key Laboratory of Materials for Energy Conversion, Department of Materials Science and Engineering, University of Science and Technology of China, Hefei 230026, P. R. China

^2^Hefei National Laboratory for Physical Sciences at Microscale, University of Science and Technology of China, Hefei 230026, P. R. China

^3^Synergetic Innovation Center of Quantum Information & Quantum Physics, University of Science and Technology of China, Hefei 230026, P. R. China

^4^National Synchrotron Radiation Laboratory, University of Science and Technology of China, Hefei 230026, PR China

^5^Laser Optics Research Center, US Air Force Academy, Colorado 80840, USA

*E-mail: fuzp@ustc.edu.cn; yllu@ustc.edu.cn


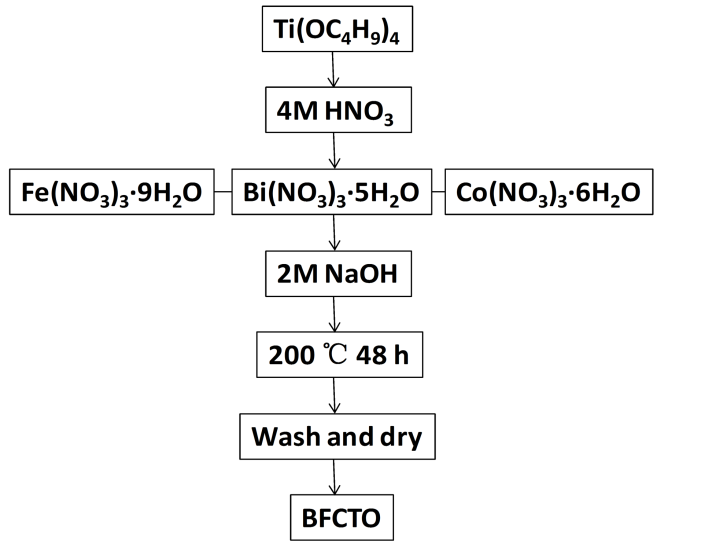


**Supplementary Figure 1** | **Typical hydrothermal thermal preparation procedure of BFCTOs**.


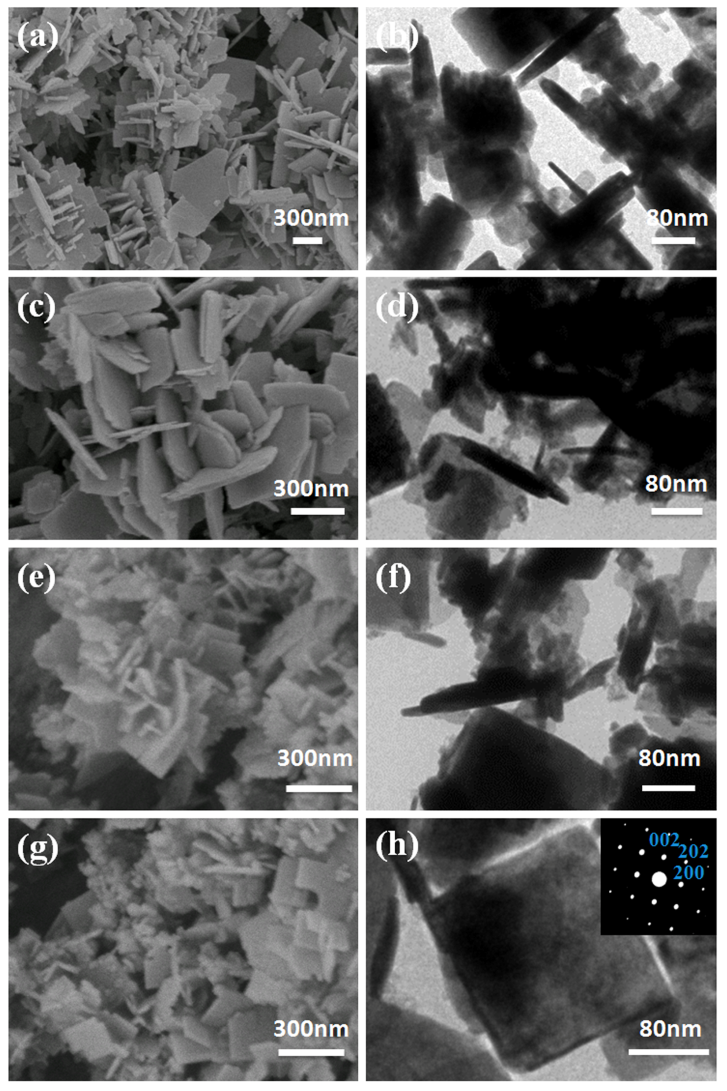


**Supplementary Figure 2 | SEM and TEM images of BFCTOs. (a, b) BFTO; (c, d) BFCTO-0.1; (e, f) BFCTO-0.2; (g, h) BFCTO-0.25.**


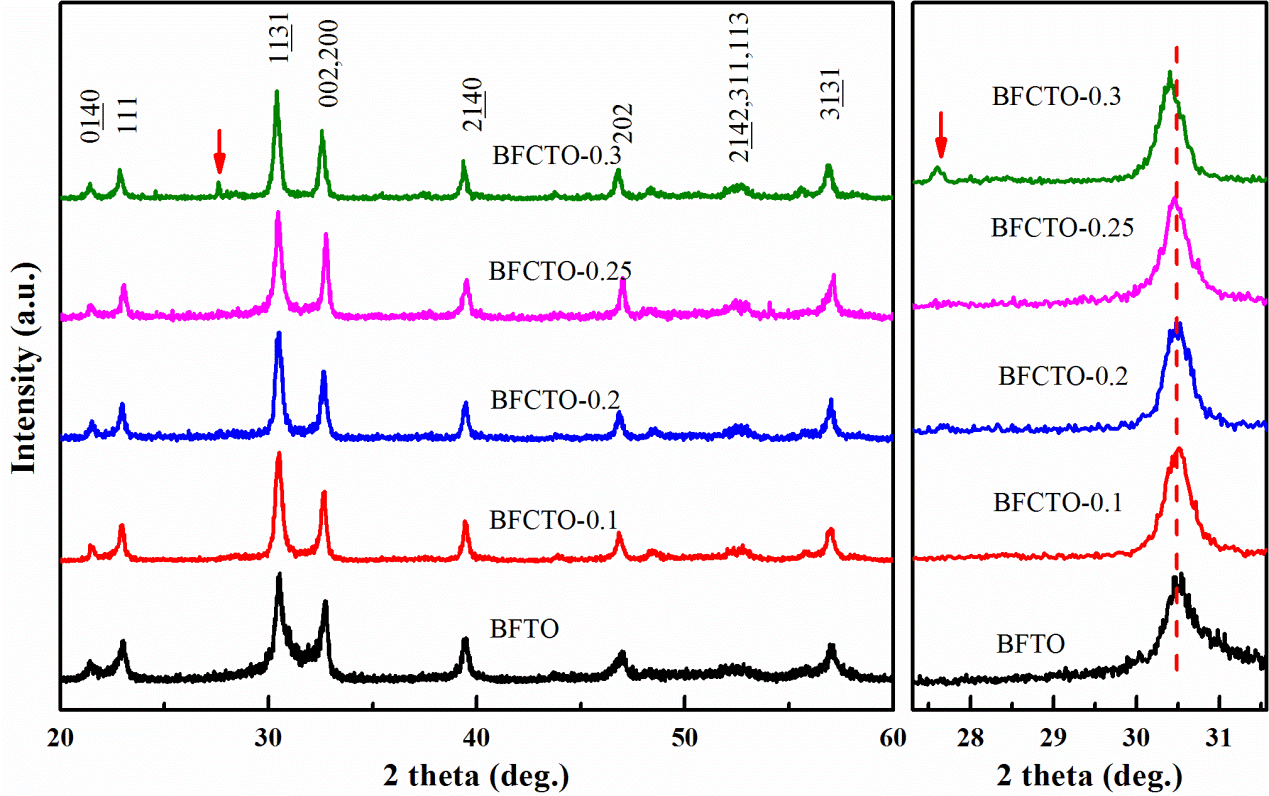


**Supplementary Figure 3 | XRD patterns of the as-synthesized BFCTOs nanoplates.
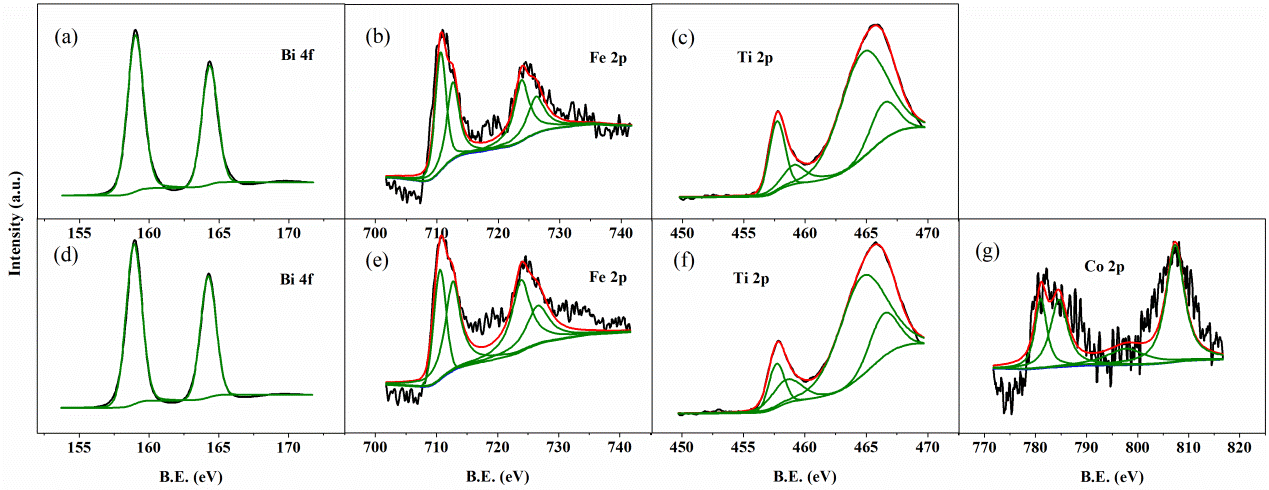
**

**Supplementary Figure 4 | XPS spectra of (a) Bi 4f, (b) Fe 2p, and (c) Ti 2p for BFTO; (d) Bi 4f, (e) Fe 2p, (f) Ti 2p and (g) Co 2p for BFCTO-0.25, respectively.**

**
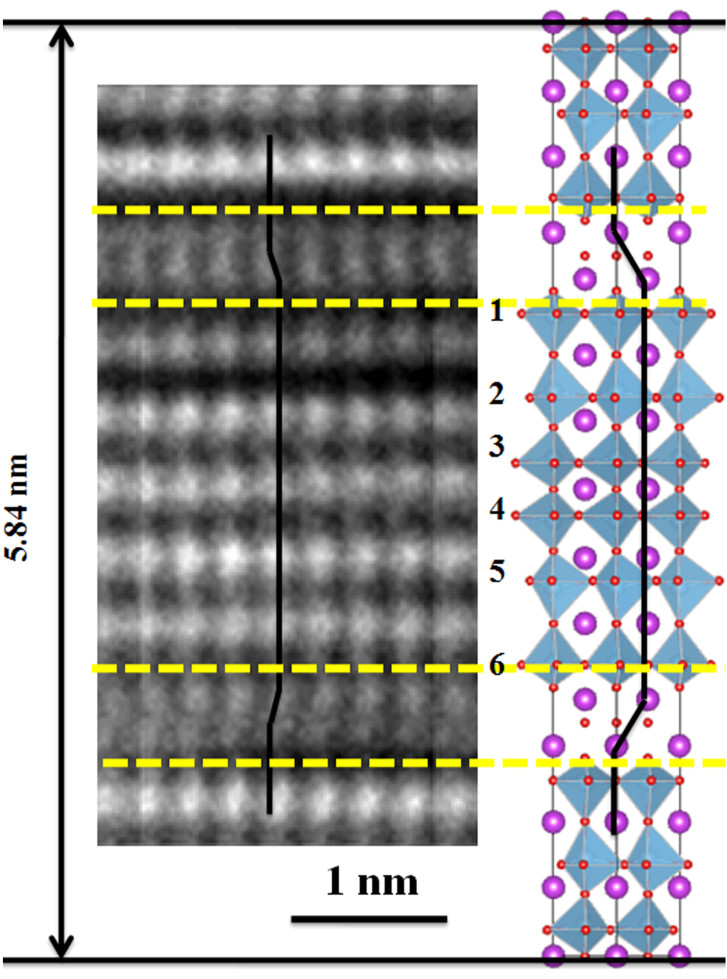
**

**Supplementary Figure 5 | Demonstration of atomic configuration picked up from HRTEM of the nanoplate-like BFCTO-0.1 (left) and calculation crystal structure of Bi_7_Fe_3_Ti_3_O_21_ (right).**


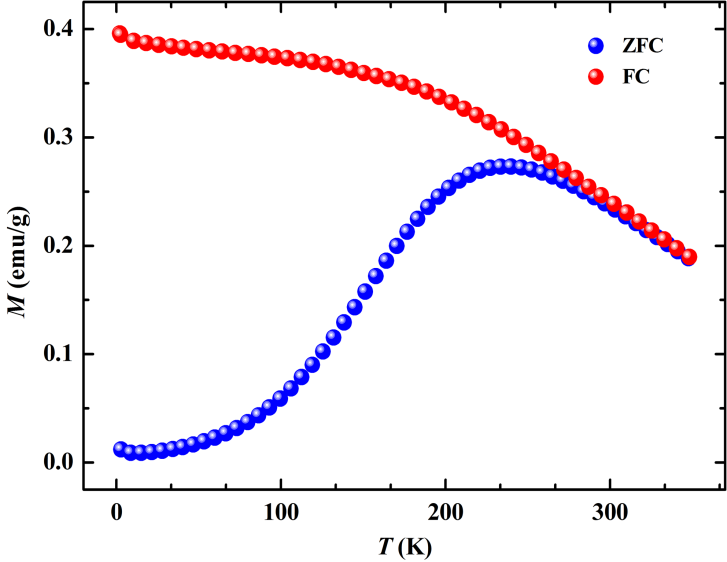


**Supplementary Figure 6 | Temperature dependence of the magnetization for the BFCTO-0.25, showing** **zero field cooling (ZFC) and field cooling (FC) curves, with an applied magnetic field set at 200 Oe.**


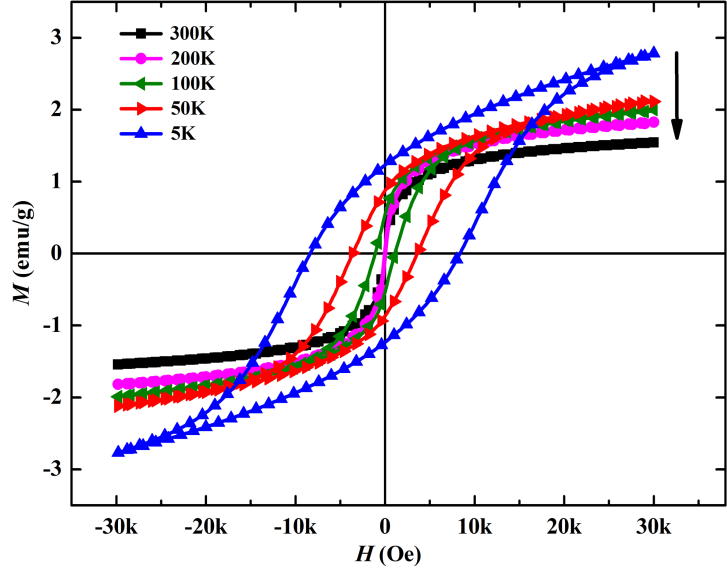


**Supplementary Figure 7 | M-H loops of BFCTO-0.25 at different temperatures from 5 K-300 K.**


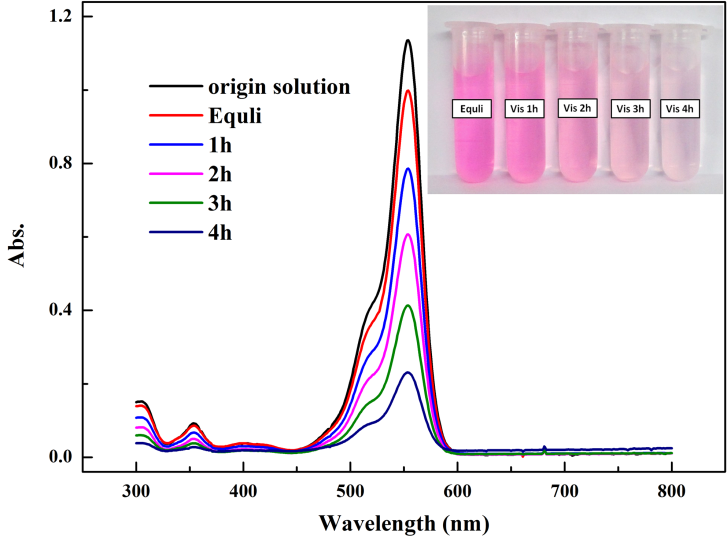


**Supplementary Figure 8 | UV-Vis spectral changes with time of RhB (5 *m*g/L) sampled from RhB/BFCTO-0.1 suspension under 20 W fluorescent lamp light (400-720 *n*m)**; inset is photographs of RhB solution after different time of visible light photocatalysis treatment.


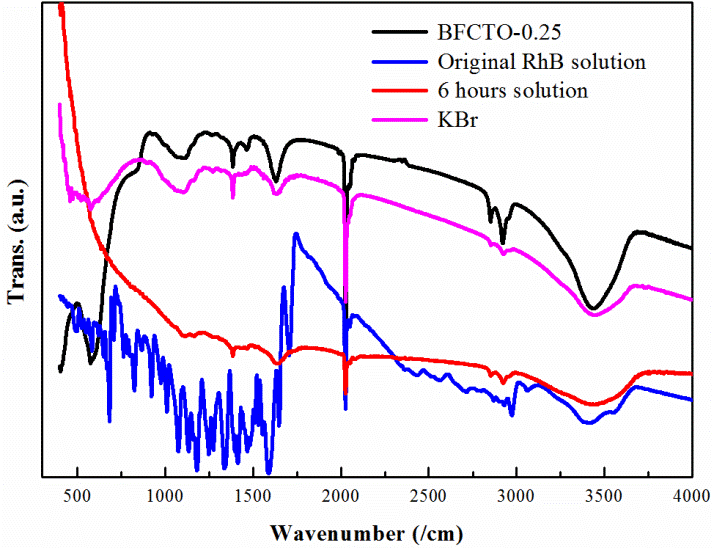


**Supplementary Figure 9 | Infrared spectroscopy** (Pressing Potassium Bromide Troche) of the final solution as well as BFCTOs powders after 6 hours photocatalytic reaction.

**Supplementary Table 1 | Binding energy of metal elements of BFTO and BFCTO-0.25 sample**

|  | BFTO | | BFCTO-0.25 | |  | BFTO | | BFCTO-0.25 | |
| --- | --- | --- | --- | --- | --- | --- | --- | --- | --- |
| Ti 2p | 457.7  464.7 | +4 2p3/2 | 457.7  464.7 | +4 2p3/2 | Fe 2p | 710.7 | +3 2p3/2 | 710.7 | +3 2p3/2 |
|  |  | +4 2p1/2 |  | +4 2p1/2 |  | 723.8 | +3 2p1/2 | 723.9 | +3 2p1/2 |
|  | 459.1  466.4 | +4 2p3/2 | 458.6  466.4 | +4 2p3/2 |  | 712.6 | +3 2p3/2 | 712.7 | +3 2p3/2 |
|  |  | +4 2p1/2 |  | +4 2p1/2 |  | 726.1 | +3 2p1/2 | 726.2 | +3 2p1/2 |
| Bi 4f | 164.3 | +3 4f5/2 | 164.2 | +3 4f5/2 | Co 2p |  |  | 781.3 | +3 2p3/2 |
|  |  |  |  |  |  |  |  | 784.7 | sat |
|  | 159.1 | +3 4f7/2 | 158.9 | +3 4f7/2 |  |  |  | 797.2 | +3 2p1/2 |
|  |  |  |  |  |  |  |  | 807.5 | sat |

Note: referenced by NIST Databases (http://srdata.nist.gov/xps/)

**Supplementary Table 2 |** **BET surface area of all prepared samples**

| Sample | BFTO | BFCTO-0.1 | BFCTO-0.2 | BFCTO-0.25 |
| --- | --- | --- | --- | --- |
| BET(*m²/g*) | 12.80 | 12.15 | 10.09 | 8.29 |

**Supplementary Table 3 | Performance of recently developed NIR responsive photocatalysts**

| **Year** | **Material** | **Light source** | **Dosage** | **Pollutant** | **Descom.** |
| --- | --- | --- | --- | --- | --- |
| 2013[^1^](#_ENREF_1) | NaYF_4_:Yb,Tm@TiO2 | 980 nm(10 W/cm^2^) | 0.5 mg | MB (0.5mL,15 mg/L) | 14h(65%) |
| 2014[^2^](#_ENREF_2) | β-NaYF_4_:Yb^3+^,Tm^3+^/Er^3+^  @SiO_2_@TiO_2_ | 980 nm(15 W/mm^2^) | 10 mg | RhB (10 mL,10^−5^ M) | 6 h(90%) |
| 2014[^3^](#_ENREF_3) | Er^3+^/Tm^3+^/Yb^3+^ doped  CaWO_4_@(TiO_2_/CaF_2_) | 980 nm(2.0 A) | 20 mg | MO(20 mL,20 mg/L) | 3h(20%) |
|  |  | 780 nm(1000 W) |  |  | 3h(48%) |
| 2014[^4^](#_ENREF_4) | BiOI/ZnWO_4_:Er^3+^, Tm^3+^ | 980 nm(2.0 A) | 40 mg | MO(40 mL,10ppm) | 3h(22%) |
| 2014[^5^](#_ENREF_5) | BiVO_4_/CaF_2_:Er^3+^,Tm^3+^, Yb^3+^ | 980 nm(2.0 A) | 20 mg | MO(10 mg/L,10 mL) | 6h(10%) |
| 2014[^6^](#_ENREF_6) | NaYF_4_:Yb, Tm/CdS/TiO_2_ | 980 nm(2.0 W/cm^2^) | 0.5 mg | MB(15mg/L,0.5 mL) | 50h(100%) |
| 2015[^7^](#_ENREF_7) | WS_2_ | 5W NIR LED(95 mW/cm^2^) | 50 mg | MO(20 mg/L^2^,50 mL) | 5h(80%) |
| 2015[^8^](#_ENREF_8) | H_x_WO_3_/WO_3_ | >760 nm(500W Xe) | 50 mg | MO(10 mg/L,50 mL) | 2h(34%) |
| 2015[^9^](#_ENREF_9) | Ag_2_O | >610 nm(500 W Xe,0.18 klx) | 30 mg | MO(10mL,16 mg/L) | 2 h(100%) |
| 2015[^10^](#_ENREF_10) | Ag_2_S | > 850 nm(500 W Xe,0.09-0.12 klx) | 100 mg | MO(10 mL,16 mg/L) | 70min(100%) |

**References：**

1. Tang, Y., Di, W., Zhai, X., Yang, R. & Qin, W. NIR-responsive photocatalytic activity and mechanism of NaYF_4_:Yb,Tm@TiO_2_ core-shell nanoparticles. *ACS Cata.* **3**, 405-412, (2013).

2. Wang, W., Huang, W., Ni, Y., Lu, C. & Xu, Z. Different upconversion properties of beta-NaYF_4_:Yb^3+^,Tm^3+^/Er^3+^ in affecting the near-infrared-driven photocatalytic activity of high-reactive TiO_2_. *ACS Appl. Mater. Inter.* **6**, 340-348, (2014).

3. Shouqiang Huang *et al.* An efficient near infrared photocatalyst of Er^3+^/Tm^3+^/Yb^3+^ tridoped (CaWO_4_@(TiO_2_/CaF_2_)) with multi-stage CaF_2_ nanocrystal formation. *J. Mater. Chem. A* **2**, 16165–16174, (2014).

4. ShouqiangHuang *et al.* Upconversion assisted BiOI/ZnWO_4_:Er^3+^,Tm^3+^,Yb^3+^ heterostructures with enhanced visible and near-infrared photocatalytic activities. *RSC Adv.* **4**, 61679-61686, (2014).

5. Huang, S. *et al.* Near-infrared photocatalysts of BiVO_4_/CaF_2_:Er^3+^, Tm^3+^, Yb^3+^ with enhanced upconversion properties. *Nanoscale* **6**, 1362-1368, (2014).

6. Guo, X. *et al.* Enhanced near-infrared photocatalysis of NaYF_4_:Yb, Tm/CdS/TiO_2_ composites. *Dalton Trans.* **43**, 1048-1054, (2014).

7. Sang, Y. *et al.* From UV to near-infrared, WS_2_ nanosheet: a novel photocatalyst for full solar light spectrum photodegradation. *Adv. Mater.* **27**, 363-369, (2015).

8. Zhang, L., Wang, W., Sun, S. & Jiang, D. Near-infrared light photocatalysis with metallic/semiconducting H_x_WO_3_/WO_3_ nanoheterostructure in situ formed in mesoporous template. *Appl. Cata. B* **168-169**, 9-13, (2015).

9. Jiang, W. *et al.* Silver Oxide as Superb and Stable Photocatalyst under Visible and Near-Infrared Light Irradiation and Its Photocatalytic Mechanism. *Ind. Eng. Chem. Res.* **54**, 832-841, (2015).

10. Jiang, W. *et al.* Photocatalytic performance of Ag_2_S under irradiation with visible and near-infrared light and its mechanism of degradation. *RSC Adv.* **5**, 24064-24071, (2015).
